# Simplified Dynamic Phantom for Pediatric Renography: A Description of Instrument and its Performance

**DOI:** 10.22038/AOJNMB.2018.11803

**Published:** 2019

**Authors:** Takashi Kamiya, Tadashi Watabe, Koichi Fujino, Romanov Victor, Yoshiki Kawamura, Kayako Isohashi, Keiko Matsunaga, Mitsuaki Tatsumi, Hiroki Kato, Eku Shimosegawa, Jun Hatazawa

**Affiliations:** 1 Division of Radiology, Department of Medical Technology, Osaka University Hospital, Suita, Japan; 2 Department of Nuclear Medicine and Tracer Kinetics, Osaka University Graduate School of Medicine, Suita, Japan; 3 Department of Molecular Imaging in Medicine, Osaka University Graduate School of Medicine, Suita, Japan; 4 Department of Radiology, Osaka University Hospital, Suita, Japan; 5 Department of Diagnostic and Interventional Radiology, Osaka University Graduate School of Medicine, Suita, Japan; 6 Immunology Frontier Research Center, Osaka University, Suita, Japan

**Keywords:** Pediatric dose guidelines, Pediatric renography, Simplified dynamic phantom, ^ 99m^Tc-MAG_3_

## Abstract

**Objective(s):**

Renography is used for the diagnostic evaluation of pediatric patients with a suspected obstruction of urinary tract or impaired renal function. The recommended dose for children have been released by the European Association of Nuclear Medicine, Society of Nuclear Medicine and Molecular Imaging, and Japanese Society of Nuclear Medicine. Since acquisition counts in dynamic scintigraphy are affected by the administered doses and sensitivity of the scintillation camera, the scan procedure should be determined independently.

In this study, we constructed simplified dynamic phantom imitating pediatric renography and tested its performance.

**Methods:**

Simplified dynamic phantom consisted of three components (i.e., infusion, imitated kidney, and drainage sections). The infusion rates (mL/min) were determined by comparing the time activity curves obtained from patients with normal renal function. The time-points of the maximum counts (T_max_), as well as the two-thirds and one-half of the maximum counts (T_2/3_ and T_1/2_) were measured in different doses using the phantom with the best-match infusion rate and duration, and low-energy general-purpose (LEGP) or low-energy high-resolution (LEHR) collimators and applying different attenuations.

**Results:**

The best-match infusion rates of the phantom to imitate the time activity curve of the normal renal function were 42.0, 1.0, 0.6, and 0.3 mL/min in the arterial, secretory, early-excretory, and late-excretory phases, respectively. When 30 MBq, LEHR collimator and non-water-equivalent phantom were applied, T_max_, T_2/3_, and T_1/2_ were 242±15.3, 220±10.0 and 317±25.2 seconds, respectively. Using LEGP collimator and (3 MBq of activity) 5-cm water-equivalent phantom, T_max_, T_2/3_, and T_1/2_ values were estimated as 242±5.8, 213±11.5, and 310±17.3 sec, respectively.

**Conclusion:**

Our simplified dynamic phantom for pediatric renography could imitate the time activity curves obtained from patients with normal renal function. T_max_, T_2/3_, and T_1/2_ could be measured under various settings of dose, collimator, and tissue attenuation.

## Introduction

Renography is used to evaluate patients with the suspected obstruction of the urinary tract or impaired renal function due to the favorable characteristics of this functional imaging modality (1-6). In a recent global survey on pediatric nuclear medicine, Fahey et al. (7, 8) indicated that the three most common procedures performed on children were ^99m^Tc bone scanning, ^99m^Tc renography, and ^99m^Tc renal cortical scintigraphy. ^99m^Tc renography is among the most commonly used examinations all over the world for adults and children to evaluate dynamic renal function. 

In accordance with the “as low as reasonably achievable” concept, the administered dose of radiopharmaceuticals should be carefully determined for pediatric patients. The Pediatric Committee of the European Association of Nuclear Medicine (EANM) published a guideline for standard and diuretic renography in children (9). In 2007, the EANM released a pediatric dosage card (10), which recommends 15 MBq of ^99m^Tc-mercaptoacetyltriglycine (MAG_3_) for pediatric patients weighing 3 kg. 

In 2011, the North American consensus guidelines published recommendations for a set of injection doses (11), depending on the radiopharmaceutical usage and body weight of pediatric patients. Furthermore, in 2014, the Japanese Society of Nuclear Medicine (JSNM) released its own guidelines (12). All of the guidelines recommend the adjustment of the administered dose according to the body weight. In addition, the JSNM guideline proposes that the scan parameters, such as the equipped collimator, scanner sensitivity, and pixel size, should also be considered for each individual pediatric study. In static or single-photon emission computed tomography examinations, the scan duration should be extended under sedation until a sufficient acquisition count is acquired. Nevertheless, given the short scan time per frame in the arterial phase, it is difficult to determine the optimal radioactivity dose of dynamic renal scintigraphy for the pediatric patients. 

The acquisition count is affected by both administered dose and scan parameters. This explains the necessity of a physical phantom in the optimization of the scan parameters for each scanner in relation to the administered dose. Several studies have investigated the use of physical phantoms for dynamic renal scintigraphy (13-15). 

Previously, physical phantoms were used in an attempt to elaborate a simulation of the renal time activity curve (TAC), which consists of arterial, secretory, and excretory phases. However, these phantoms were too complex to use in every hospital. Therefore, it is essential to construct a simplified dynamic phantom to evaluate the sensitivity of a scanner easily. With this background in mind, the present study was conducted to create a simplified dynamic phantom imitating pediatric renography and test its performance. 

## Methods

***Structure of the simplified dynamic phantom for pediatric renography***

**Figure 1:**
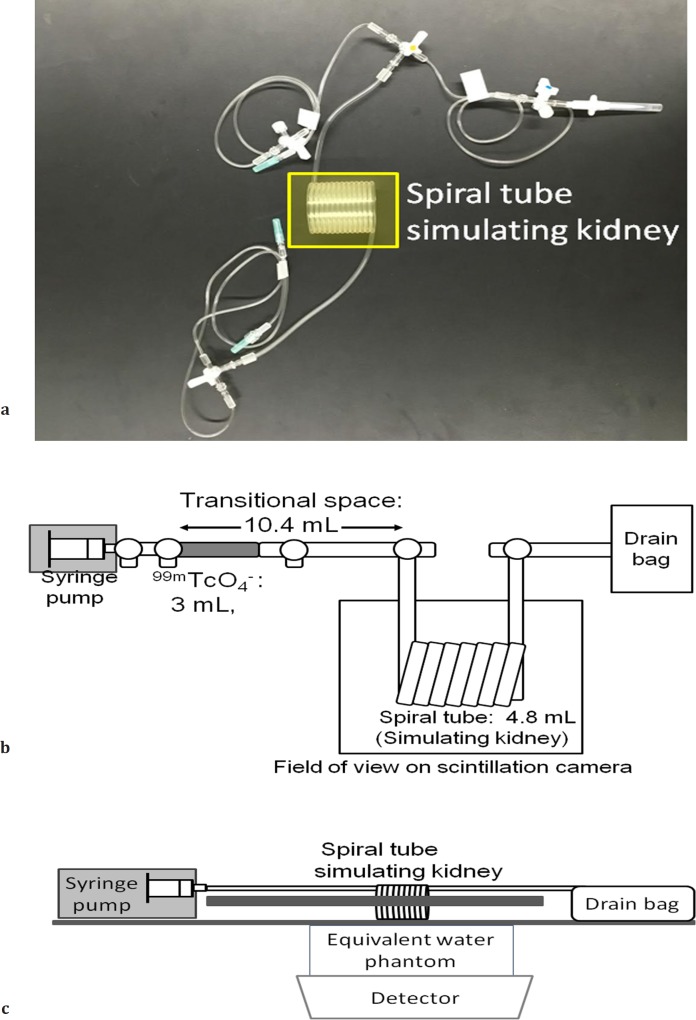
Photo (a) and overviews (b: top and c: side) of the simplified dynamic phantom for pediatric renography

The phantom consisted of three components, namely infusion, kidney-simulation, and drainage sections (Figure 1). The tracer delivery system used in this study was comprised of a 50-mL syringe (SS-50LZ; TERUMO), equipped with the advance syringe pump (PHD2000-P; Harvard Apparatus). This pump has programmable configurations of infusion. A 50-mL syringe was connected to three-way cocks (Discofix; B. Brawn) to fill the syringe with tap water. Another end of the three-way cock was connected to three plastic tubes (SF-ET3825L [3.8 mL]: TERUMO), which were simulated for transitional space. 

The kidney of the phantom was simulated by a spiral tube (Disposable infusion set; Universal Giken) with a size of 4.5×4.5×5.0 cm^3^ and inner volume of 4.8 mL. One end of the disposable infusion set was connected to the infusion part, and another end was linked to the drainage section, which consisted of the waste bag with plastic tubes and three-way cocks. The spiral tube of the disposable infusion set was fixed to polyurethane foam (Dow Chemical Styrofoam) to prevent the motion during the scan procedure. 

All parts of the phantom were comprised of commercially available and commonly used components. The equivalent solid phantom for water (Solid Water, Toyo Medic, Tokyo, Japan) was placed between the phantom and detector to simulate the soft tissue for attenuation.

***Preparation of phantom and scan protocol***

All tubes were filled with tap water, and a solution of ^99m^TcO_4_^-^ was mixed with dye to track the flow in the plastic tube. The spiral tube section of the phantom was placed at the center of the field of view of the scintillation camera. Subsequently, the scan acquisition was started simultaneously with the infusion using the syringe pump. 

A scintillation camera (Bright View, Philips) equipped with a low-energy general-purpose (LEGP) collimator or a low-energy high-resolution (LEHR) collimator was used for this study to compare the acquisition counts. The data were acquired in a 128×128 matrix in the dynamic mode (pixel size: 4.66 mm, total scan duration: 20 min; 1 sec ×80 frames, 10 sec ×112 frames). 

***Clinical renal model***

A clinical renal model was created as a standard reference for the optimization of the infusion parameters of the phantom. Due to the lack of any previous reports on normal pediatric renal scintigraphy, the clinical renal model was created based on young healthy donors. To this end, the data of six young healthy donors for renal transplantation (i.e., 6 males with the mean age of 25.3±2.9 years and age range of 21-29 years) who underwent dynamic renal scintigraphy at Osaka University Hospital from April 2000 to April 2014 were used to generate the clinical renal model. 

Dynamic renal scintigraphy was performed with the bolus injection of ^99m^Tc-MAG_3_(262.3±7.7 MBq). The scintillation camera (E.CAM, Siemens) was equipped with LEHR collimator for clinical dynamic renal scintigraphy. The data were acquired in a 128×128 matrix in the dynamic mode (pixel size: 3.90 mm, 1 sec ×80 frames, 10 sec ×112 frames; total scan time: 20 min). The regions of interest were drawn on both kidneys and backgrounds. The acquisition count was normalized to each maximum count. 

***Infusion rates of simplified dynamic phantom***

The infusion duration and rate of the programmable syringe pump were adjusted relative to the time activity curve of the clinical renal model. The infusion dose was set at 30 MBq in order to obtain the accurate renal parameters of the phantom. The clinical renal model consisted of four phases, namely arterial, secretory, early-excretory, and late-excretory.

Following the previous studies (13-16), the infusion durations of each phase were fixed at 10, 420, 120, and 650 sec. The first infusion rates for arterial phase varied between 30 and 60 mL/min for the estimation of the inflection point, which is defined as the transition point between arterial phase (i.e., rapid linear increase) and secretory phase (i.e., gradual increase). Furthermore, the second, third, and fourth infusion rates varied between 0.1 and 2.0 mL/min for the estimation of the T_max_, T_2/3_, T_1/2_, respectively. 

The T_max_ value was the time of the maximum count. The T_2/3_ and T_1/2_ values were the time-points when two-thirds and one-half of the maximum radioactivity were reached. In order to adjust the infusion parameters, the acquisition counts of the phantom were normalized to the maximum count of each acquisition.

***Comparison of renal parameters with dose, collimators, and tissue attenuation***

The minimum administered dose of ^99m^Tc-MAG3 recommended by EANM guidelines is 15 MBq for the pediatric patients weighing 3 kg. According to the previous reports (17-20), the extraction rate of the radiopharmaceuticals was considered to be 40% in both kidneys, and infusion dose into the phantom was set at 3 MBq. The thickness of equivalent solid phantom was set at 1, 3, and 5 cm to simulate the attenuation thickness of a pediatric patient’s soft tissues between the phantom and detector. 

The scan procedure under each condition was repeated three times. The T_max_, T_2/3_, and T_1/2_ values, and average acquisition counts between 60 and 120 sec simulated in accordance with the pediatric dose guidelines were compared between the LEGP and LEHR collimators. 

***Statistical Analysis***

The data were expressed as mean and standard deviation. The Tukey’s test and Student’s t-test were used to test the statistical difference. The comparison of collimators was accomplished by using paired sample t-test. All statistical tests were performed in Excel 2016. P-value less than 0.05 was considered statistically significant.

## Results

**Figure 2:**
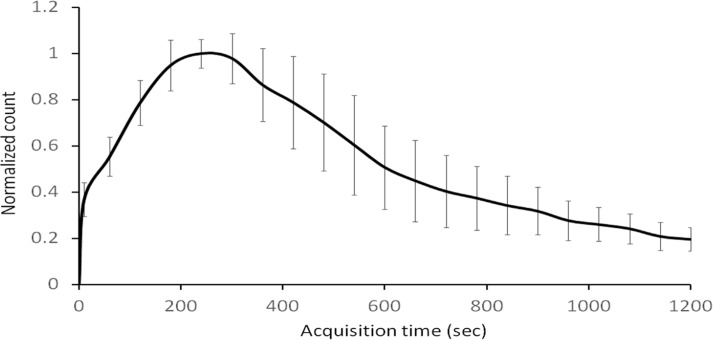
Mean time activity curve of the normal renal model

**Figure 3:**
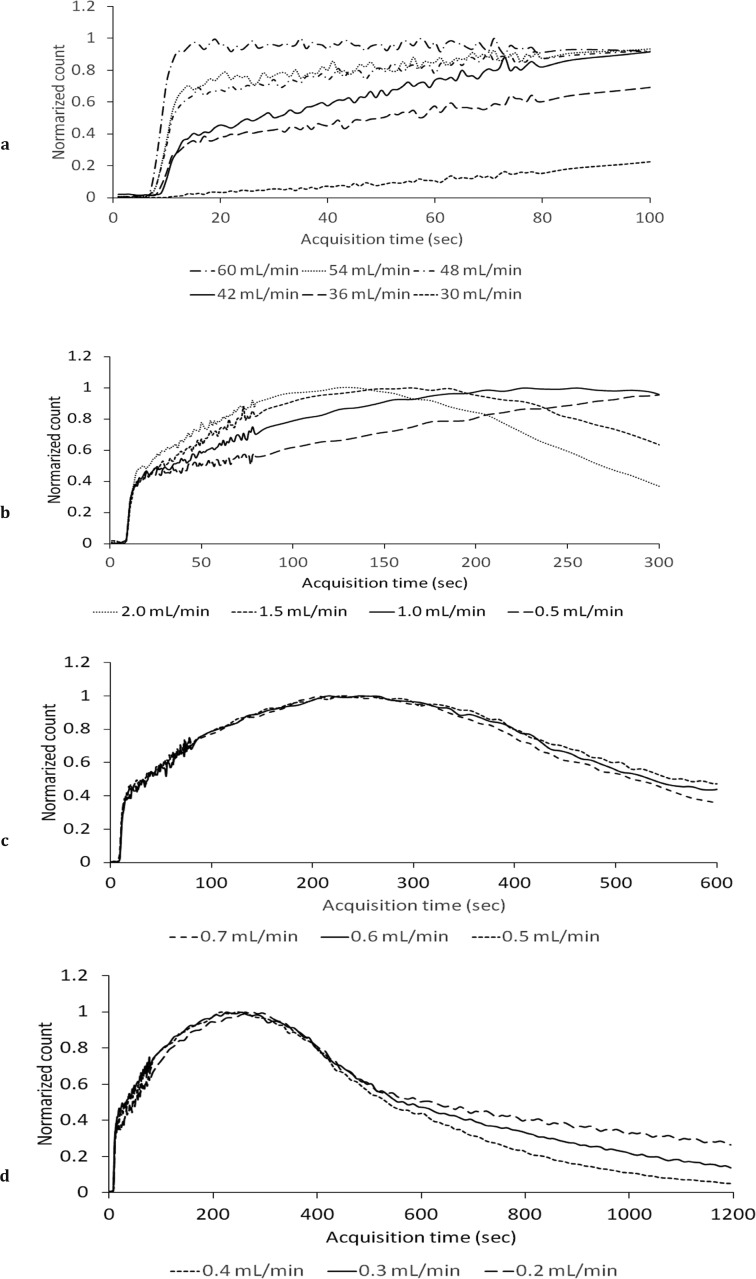
Time activity curves of simplified dynamic phantom for pediatric renography

Figure 2 illustrates the clinical renal model created based on young healthy donors for renal transplantation. The normalized count at the inflection point (10 sec after the scan initiation) was 0.37±0.072. The mean T_max_, T_2/3_, and T_1/2_ values were 275±87.2, 192±91.1, and 341±130 sec, respectively. Figure 3 depicts the TACs of the phantom with various infusion rates. The best-match infusion rate for the first phase was determined as 42 mL/min considering its normalized count (0.40) at the inflection point, which showed the best-match to those of the normal TAC (0.37±0.072). 

**Figure 4:**
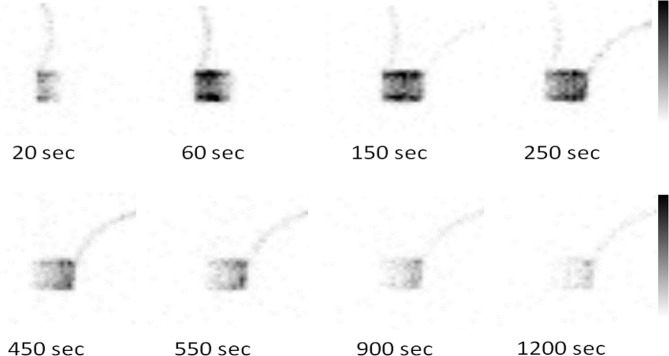
Dynamic images of the simplified dynamic phantom

^99m^TcO_4_^-^ solution passage through the phantom is shown from the upper left to the lower right corner. The scan duration of each image was 10 sec, and the infusion dose was 30 MBq. LEHR collimator and non-water-equivalent phantoms were applied

Similarly, the best-match infusion rates for the second, third, and fourth phases were determined as 1.0, 0.60, and 0.30 mL/min, respectively. Table 1 presents the best-match infusion parameters of the phantom. The mean T_max_, T_2/3_, and T_1/2_ values of the normal renal model were 242±15.3, 220±10.0, and 307±28.9 sec, respectively (Table 2). Figure 4 displays the images of the phantom in each phase.

**Figure 5:**
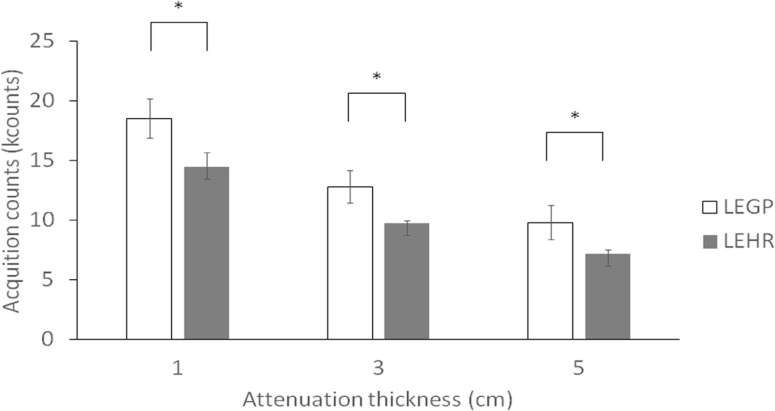
Total acquisition counts from 60 to 120 seconds under the guidelines

Under the pediatric dose guidelines, the integration of acquisition counts between 60 and 120 sec is shown in Figure 5. Acquisition counts were significantly smaller in the LEHR collimator than in the LEGP collimator. The reduction rates of acquisition counts between the LEHR and LEGP collimators with 1, 3, and 5-cm simulated thicknesses were 33.8±1.9%, 32.4±6.2%, and 34.0±8.8%, respectively. 

**Figure 6:**
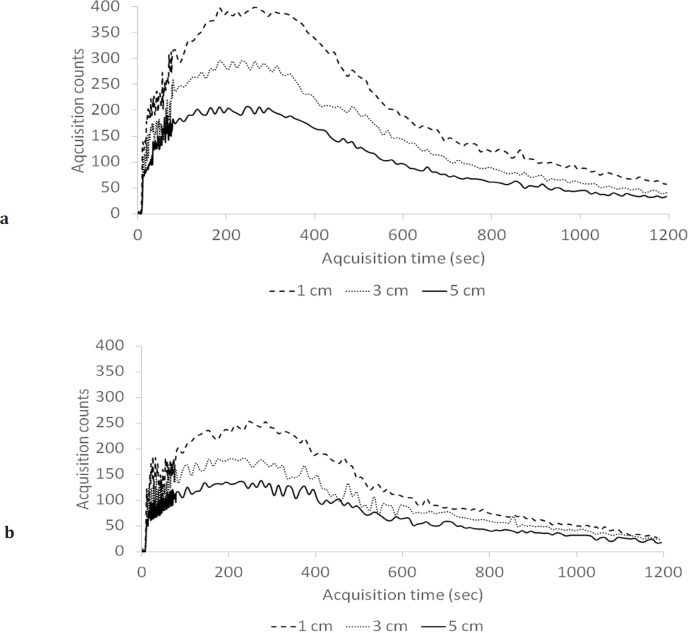
Time activity curves of the simplified dynamic phantom under the guidelines

**Figure 7:**
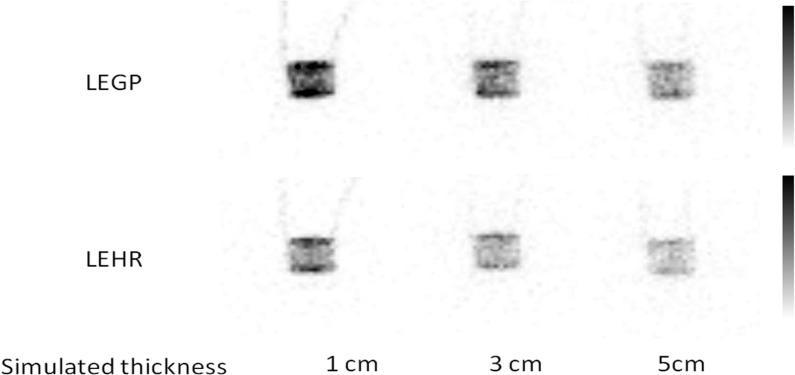
Planar images of the phantom under the guidelines

The decreased count ratios of LEGP and LEHR by attenuation thickness were 2.1 and 1.5 kcounts/cm, respectively. Figure 6 presents the TACs of LEGP and LEHR collimators. The T_max_, T_2/3_ and T_1/2_ values of each condition simulated under the guidelines are shown in Table 2. There was no significant difference in these parameters among the different conditions of the collimators and simulated thickness. The images of the simplified dynamic phantom at maximum acquisition count are shown in Figure 7. 

## Discussion

This study was targeted toward the construction of a simplified dynamic phantom imitating pediatric renography with reference to the clinical renal model and examination of the phantom performance. The coefficient of variation (CV) of the T_max_ in our phantom was 6.3%, which is small enough, compared to the values reported by the previous dynamic phantom studies (13-16). In previous studies on the clinical normal renal function, the T_max_ and T_1/2 _values were reported as 192±33 and 320±73 sec, which are consistent with our results (17-20). 

In the current study, the thickness of the equivalent solid phantom for attenuation ranged from 1-5 cm. This thickness was decided based on the computed tomography (CT) data of 10 pediatric patients with a body weight of < 3 kg in our hospital (mean age 33.5±67.9 days, age range: 1-222 days). Based on the CT images, the maximum distance between the kidney and back skin was calculated as 3.23 cm (mean thickness: 2.07±0.46 cm). 

**Table 1:** Infusion durations and rates of the phantom

**Infusion phase**	**Infusion duration (sec)**	**Optimized infusion rate (mL/min)**
1 (arterial)	10	42
2 (secretory)	420	1.0
3 (early excretory)	120	0.60
4 (late excretory)	650	0.30

**Table 2:** Renal parameters of the phantom

**Collimator**	**Simulated thickness (cm)**	**T**_max_ ** (sec)**	**T**_2/3_ ** (sec)**	**T**_1/2_ ** (sec)**
LEHR	0	242 ± 15.3	210 ± 10.0	307 ± 28.9
**(infusion dose: 30 MBq)**				
	1	242 ± 20.8	213 ± 5.8	340 ± 20.0
LEGP	3	252 ± 15.3	217 ± 25.2	313 ± 25.2
	5	242 ± 5.8	213 ± 11.5	310 ± 17.3
	1	242 ± 15.3	233 ± 11.5	293 ± 11.5
LEHR	3	248 ± 11.5	210 ± 10.0	303 ± 23.1
	5	245 ± 26.5	207 ± 20.8	307 ± 28.9

The phantom consisted of three components (i.e., infusion, kidney-simulation, and drainage sections) and was assembled using commonly used and easily accessible materials. The spiral tube set corresponded to the kidney-simulation in this phantom. The water-equivalent phantoms were simulated for the attenuation of soft tissue

Mean time activity curve of the normal renal TAC model which was obtained from six young healthy donors for renal transplantation

Time activity curves of the phantom at the first infusion rate for arterial phase (a), second infusion rate for secretory phase (b), third infusion rate for early excretory phase (c), and fourth infusion rate for late-excretory phase (d). Infusion dose was 30 MBq. LEHR collimator and non-water-equivalent phantoms were applied

There was a significant difference between LEGP and LEHR collimator in terms of the total counts under the guidelines. (*: P<0.05 by paired sample t-test)

Time activity curves of the phantom using (a) LEGP collimator, (b) LEHR collimator with different thicknesses of equivalent solid phantoms for water

Planar images of the phantom acquired in accordance with the guidelines at maximum acquisition counts

The acquisition counts with LEHR collimator and attenuation thickness of 5 cm showed approximately 40% decrease in comparison with those of LEGP collimator with 1-cm attenuation thickness. However, the renal parameters (i.e., T_max_, T_2/3,_ and T_1/2_) displayed no significant difference in this regard. Although the guidelines recommend the use of LEGP collimator in pediatric renography, our phantom study suggested that the LEHR is also applicable.

Several phantoms for renography were previously reported by other researchers. Heikkinen et al. constructed an automated physical phantom for renography (13). They simulated clinical conditions with various renal functions considering the renal and heart flow, attenuation, and scatter from several organs or background using containers. Accordingly, they obtained a good reproducibility with a maximum CV of 1.3% for the T_max_ value. Nykanen et al. evaluated and standardized dynamic radionuclide protocols through a multicenter study of renography using the Heikkinen phantom in Finland (16). 

Ahmed et al. constructed a physical dynamic phantom using Plexiglas boxes (14). The maximum CV of the T_max_ value in the Ahmed phantom was 11.8%. They simulated not only the circulation of the kidney, heart, liver, and bladder, but also physiologic blood pressure. Both of the phantoms created by Heikkinen and Ahmed included right and left kidneys and other organs. The images of these phantoms have been reported to be similar to those of real patients. However, these physical phantoms are too complex for clinical practice. 

Our simplified dynamic phantom for pediatric renography is simpler than the mentioned phantoms. Nonetheless, our phantom is enough for the evaluation of the scan parameters before each procedure. This simplified dynamic phantom can be constructed with commonly used and easily accessible materials, which facilitate its broad application in many institutions. Furthermore, similar phantoms could be constructed to optimize the appropriate dynamic scan protocol in pediatric patients. 

The advantage of our phantom is that we can easily modify the infusion rate and duration of the syringe pump injection. These parameters can be changed to simulate low-clearance conditions, such as renal insufficiency, hydronephrosis, and renal artery stenosis. On the other hand, this study contains several limitations. The phantom was constructed without taking the scatter by other organs into consideration. However, the ratios of the radioactivity concentrations between the background and both kidneys were below 0.05 in previously reported phantoms (14, 15). The effect of the scatter is considered negligible in the evaluation of image quality. 

In addition, the shape of our phantom differs from the actual kidney shape. However, it is enough for the evaluation of the relationship between the administered dose and the image quality. It is recommended to perform further studies to validate the scan parameters defined by our phantom by evaluating the image quality of dynamic renal scintigraphy in pediatric patients.

Minimization of the radiation exposure from radiopharmaceuticals, especially in pediatric patients, is as important as the maintenance of the diagnostic quality of dynamic renal scintigraphy regarding the renal diseases (21-27). The Nuclear Medicine Global Initiative (NMGI), comprising 13 international organizations, recommended activity doses for pediatric examinations (7, 8). 

According to the NMGI, renography accounts for about 20% of all pediatric radioisotope studies and represents the second most commonly performed study in the pediatric age group. Consequently, it would be advantageous to use a simplified dynamic phantom to decide the optimal scan parameters of dynamic renal scintigraphy in pediatric patients. 

## Conclusion

The simplified dynamic phantom for pediatric renography was constructed combining several commonly used and easily accessible components. Our constructed phantom could imitate time activity curves obtained from patients with normal renal function. T_max_, T_2/3_, and T_1/2_ could be measured under various settings of dose, collimator, and tissue attenuation. This phantom might assist the determination of the appropriate activity and scan parameters, which are used for pediatric patients with normal and impaired renal function.

## Data Availability

The datasets generated and analyzed during the current study are available by the corresponding author on a reasonable request.

## Financial declaration

This study was not financially supported by any organizations.
